# Collectanea Medica

**Published:** 1813-11

**Authors:** 


### COLLECTANEA MEDICA,

CONSISTING OF

ANECDOTES, FACTS, EXTRACTS, ILLUSTRATIONS, QUERIES, SUGGESTIONS, &c.

RELATING TO THE

*History or the Art of Medicine, and the Auxiliary Sciences.*

*Additional Remarks on the State in which Alcohol exists in fermented Liquors. By William Thomas Brande, Esq. F.R.S. From the Philosophical Transactions. Read before the Royal Society, December* 17, 1812.

THE experiments and observations contained in this paper, are intended as supplementary to a communication on the same subject, which the Royal Society has done me the honor to insert in the Philosophical Transactions for the year 1811.*

On that occasion, I endeavored to refute the commonly received opinion respecting the *production* of alcohol during the distillation of fermented liquors, by showing, that the results of the process are not affected by a variation of temperature equal to twenty degrees of Fahrenheit's scale; that is, that a similar quantity of alcohol is afforded by distilling wine at 180° and at 200°.

I also conceived, that any new arrangement of the ultimate elements of the wine, which could have given rise to the formation of alcohol, would have been attended with

* P. 337.

other other symptoms of decomposition; that carbon would have been deposited, or carbonic acid evolved, which in the experiments alluded to, was not the case. Upon such grounds I ventured to conclude, that the relative quantity of alcohol in wines, might be estimated by submitting them to a careful distillation, and by ascertaining the specific gravity of the distilled liquor with the precautions which I have formerly described.

This conclusion may be objected to, by supposing that the lowest temperature, at which the distillations were performed, was sufficient for the formation of alcohol from the elements existing in the wine; but it is not easy to conceive how this should happen, without some of those other changes which I have just noticed.

It has been stated, in my former paper, that the separation of alcohol from wine, by the addition of subcarbonate of potash, is prevented by the combination of the alkaline salt with the coloring-extractive, and acid contained in the liquor. I have also shortly noticed some unsuccessful attempts to separate these substances by other means than distillation.

In prosecuting the inquiry, this difficulty has been surmounted, and I shall proceed to show, that alcohol may be separated from wine without the intervention of heat, and that the proportion thus afforded is equal to that yielded by distillation.

When the acetate,* or subacetate† of lead, or the subnitrate of tin‡ are added to wine, a dense insoluble precipitate is quickly formed, consisting of a combination of the metallic oxide, with the acid and coloring-extractive matter of the wine; and when this is separated by filtration, a colorless fluid is obtained, containing alcohol, water, and a portion of the acid of the metallic salt, provided the latter has not been added in excess, in which case a part remains undecomposed.

The acetate of lead and the subnitrate of tin produce the desired effect of separating the coloring and acid matters, in the greater number of instances, but they are less rapid and perfect in their action, and not so generally applicable as

* Sugar of lead.

† Formed by boiling two parts of sugar of lead with one of finely powdered litharge in six parts of water. The solution should be preserved in well-closed phials, as it is rapidly decomposed by attracting carbonic acid from the atmosphere. Even while hot, a portion of carbonate of lead is formed in it.

‡ Prepared by dissolving protoxide of tin in cold dilute nitric acid.

the subacetate of lead,* which is the substance that I commonly employed.

The following experiment was made with a view to ascertain the effect of this salt.

Twenty measures of alcohol, specific gravity ,82500, were mixed with eighty measures of distilled water colored with logwood, and rendered slightly acid by supertartrate of potash. Four measures of a concentrated solution of the subacetate of lead were added to this mixture, and the whole poured upon a filter. A precipitate was thus collected of a deep purple color, which appeared to consist of oxide of lead combined with tartaric acid and the coloring-extractive matter.

The filtered liquor was perfectly transparent and colorless, and afforded, on the addition of subcarbonate of potash, 19,5 measures of alcohol.†

Finding that the separation of alcohol by subcarbonate of potash from mixtures of spirit and water, was nearly complete, and that coloring-extractive matter, and tartaric acid might be removed from such mixtures by the subacetate of lead, I proceeded to examine wine by such modes of analysis.

The following results were obtained by these, and other comparative experiments.

1. One part by measure of a concentrated solution of subacetate of lead, was added to eight measures of common

* The effect of this salt upon coloring matter, was first pointed out to me by Mr. E. M. Noble, of Chelsea.

† Pure subcarbonate of potash, obtained by igniting the carbonate, was employed in these experiments. I found that about 19,5 parts of alcohol were separated in the course of four hours, by the addition of 50 parts of the subcarbonate to a mixture of 20 parts of alcohol by measure with 80 of distilled water, and that no further separation took place. The alcohol is always slightly alkaline, probably from containing a small portion of the solution of the subcarbonate, or of pure soda; but as this did not interfere with the object of the experiment, it was not particularly attended to.

When the subcarbonate was added to a mixture of four parts by measure of alcohol with 96 of water, no separation was effected.—A mixture containing 8 per cent. of alcohol afforded about 7 parts—one containing 16 per cent. about 15,5, and where the proportion of alcohol exceeded 16 per cent. the quantity, indicated by the action of the subcarbonate, was always within 0,5 per cent. of the real proportion contained in the mixture So that in the examination of wines containing less than 12 per cent. of alcohol, the method described in the text is somewhat exceptionable. The above experiments were made in glass tubes varying in diameter from 0,5 inch to 2 inches, and accurately graduated into 100 parts.

port port wine: the mixture having been agitated for a few minutes, was poured upon a filter. The filtrated liquor was perfectly colorless, and the addition of dry subcarbonate of potash effected a rapid separation of alcohol.*

100 measures of the wine thus treated, afforded 22,5 measures of alcohol.

2. Eight ounces of the wine employed in the last experiment, were distilled in glass vessels, as described in my former paper. The specific gravity of the distilled liquor at the temperature of 60° was 0,97530, which indicates 22,30 per cent. by measure of alcohol of the specific gravity of ,8250.

3. Eight ounces of the same wine were introduced into a retort placed in a sand heat, and the process of distillation was stopped when six ounces had passed over into the receiver. After the vessels were completely cooled, the portion in the receiver was added to the residuum in the retort. The specific gravity of this mixture (ascertained with proper precautions) was ,9884, that of the original wine = 0,9883.†

When care was taken to prevent the escape of vapor, no change of specific gravity was produced in the wine by three repetitions of the above process.

Similar experiments were repeated upon Madeira, Sherry, Claret, and Vin de grave, wines differing in the relative proportions of alcohol, coloring matter, and acid which they contain, and the results were as decisive; so that I conceive it is amply proved, by experimental evidence, that no alcohol is *formed* during the distillation of wines, and that the whole quantity found, after distillation, pre-existed in the fermented liquor.

It has been frequently asserted, that a mixture of alcohol and water, in the proportions I have stated them to exist in wine, would be much more effectual in producing intoxication, and the general bad effects of spirituous liquors, than a similar quantity of the wine itself. But this is true to a very limited extent only: when brandy is added to water, it is some time before the two liquids perfectly combine, and with alcohol this is more remarkably the case, and these mixtures are warmer to the taste, and more heating, if taken in this state of imperfect union, than when sufficient time has been allowed for their perfect mutual penetration.

I have also ascertained that distilled port wine tastes

---

* When any excess of the subacetate had been employed, a portion of carbonate of lead was thrown down; but this did not interfere with the subsequent separation of the alcohol.

† This experiment was suggested in the Edinburgh Review for November, 1811.

 stronger, stronger, and is more heating than the wine in its original state, and that these qualities are impaired, and the wine reduced nearly to its original flavor, by the addition of its acid and extractive matter. With claret, and some other wines, containing less alcohol and more acid than port, these circumstances are more readily perceived; and lastly, if the residuum afforded by the distillation of 100 parts of port wine, be added to 22 parts of alcohol and 88 of water (in a state of perfect combination), the mixture is precisely analogous in its intoxicating effects to port wine of an equal strength.

In the table annexed to my former paper, it appears that the average quantity of alcohol contained in port wine amounts to 23,48 per cent.; but two of the wines there alluded to are stronger than any I have since met with, and were, at that time, sent to me as "remarkably strong and old port." I have lately examined a number of specimens of the better kinds of port wine in common use, and the results of these experiments lead me to place the average strength at 22 per cent. of alcohol by measure.

A port wine procured for me by Dr. Baillie, and to which no brandy had been added, afforded 21,40 per cent. of alcohol: another specimen of a similar description, put into my hands by an Oporto merchant, contained only 19 per cent.; it is the weakest port wine I have met with.

The other results given in the table, agree perfectly with those of subsequent and more extended experiments.

---

*Observations on Radiant Heat.* By F. Delaroche, M.D.*

I propose in this memoir to state several propositions which appear to me capable of throwing some light on the theory of radiant heat; and which, I think, I have established by decisive experiments. These experiments, indeed, were made with sufficient care to prevent any doubts about their exactness; but I may be deceived in the conclusions that I deduce from them. In that case I shall readily acknowledge my error; nor shall I think that I have lost my labor if I draw, upon so interesting a subject, the attention of some philosopher more fortunate than myself, or better situated for examining it with accuracy.

First Proposition.—*Invisible Radiant Heat may in some circumstances pass directly through Glass.*

Different philosophers, and particularly Mr. Leslie, conceive that they have proved the falsehood of this proposition; but the experiments of Professor Prevost, of Geneva, have lately established its truth in an incontestable manner. He

* Abridged from the *Journal de Physique*, vol. lxxv. p. 201.

obtained obtained his result by separating the immediate effects of transmitted heat from those produced by the heating of the glass, by a process equally simple and ingenious; namely, by employing moveable screens of glass, which he renewed continually, and of course did not give them time to become heated. I have myself, since I became acquainted with the memoir of M. Prevost, made a great many experiments, which appear to me to prove the same thing. The nature of these will be stated in support of the second proposition.

Second Proposition.—*The Quantity of Radiant Heat which passes directly through Glass is so much greater relative to the whole Heat emitted in the same Direction, as the Temperature of the Source of Heat is more elevated.*

Dr. Delaroche shows, in the first place, by some ingenious experiments, that a thermometer (of the temperature of the surrounding atmosphere) exposed to the action of radiant heat rises in a given time, cæteris paribus, a quantity proportional to the rays which it receives. If a certain number of rays make it rise 1 degree, double the number will make it rise 2 degrees, triple the number 3 degrees, and so on. This being established, he placed two parabolic metallic mirrors at the distance of rather more than three feet from each other. In the focus of one mirror he placed a thermometer; and in the focus of the other a hot body, gradually increasing in temperature. The thermometer was allowed to rise to its maximum without any screen. Then a screen of transparent glass was interposed, and the experiment repeated. Lastly, a screen of blackened glass was interposed, and the experiment repeated a third time. By the blackened screen all the radiant heat would be intercepted, and the effect on the thermometer would be owing to the rise in the temperature of the screen. Hence the rise of the thermometer, when the blackened screen was used, subtracted from the same rise when the transparent screen was used, leaves the effect produced by the radiant heat passing through the glass. The following table shows the ratio between the rays passing through the clear glass, and the rays acting on the thermometer, when no screen was interposed, at different temperatures:—

| Temperature of the Hot Body in the Focus. | Rays transmitted through the Glass Screen. | Total Rays. |
|---|---|---|
| 357° | 10 | 263 |
| 655 | 10 | 139 |
| 800 | 10 | 75 |
| 1760 | 10 | 34 |
| Argand's lamp without its chimney | 10 | 29 |
| Ditto, with glass chimney | 10 | 18 |

Third

Third Proposition.—*The Calorific Rays which have already passed through a Screen of Glass, experience, in passing through a second Glass Screen of a similar nature, a much smaller Diminution of their Intensity than they did in passing through the first Screen.*

This proposition was proved by interposing, first one glass screen, and then two, and observing the difference in the effect. The experiments appear conclusive.

Fourth Proposition.—*The Rays emitted by a hot Body differ from each other in their faculty to pass through Glass.*

This proposition is an obvious consequence from the third, which can hardly leave a doubt that the calorific rays (like those of light) are of different kinds.

Fifth Proposition.—*A thick Glass, though as much or more permeable to Light than a thin Glass of worse Quality, allows a much smaller Quantity of Radiant Heat to pass. The Difference is so much the less as the Temperature of the radiating Source is more elevated.*

This proposition is proved by experiments made with glasses of different thickness employed as a screen; and its truth is sufficiently established, if these experiments be accurate. This curious fact, that radiating heat becomes more and more capable of penetrating glass, as the temperature increases, till at a certain temperature the rays become luminous, leads to the notion that heat is nothing else than a modification of light, or that the two substances are capable of passing into each other.

Sixth Proposition.—*The quantity of Heat which a hot Body yields in a given time by Radiation to a cold Body situated at a distance, increases, cæteris paribus, in a greater Ratio than the excess of Temperature of the first Body above the second.*

This proposition being at variance with the opinion of Mr. Leslie, and of several other philosophers, Dr. Delaroche thought it necessary to establish it by a great variety of experiments. These experiments leave no doubt of the fact; though they are not sufficient to enable us to deduce the rate at which the increase takes place.

I must acknowledge that this proposition appears to me somewhat puzzling. One is at first sight disposed to account for it by the inaccuracy of the thermometer as a measure of heat; but Dr. Delaroche, aware of such an objection, has remarked, that if we adopt Mr. Dalton's opinion with respect to the thermometer, so far from removing the apparent anomaly, it would only serve to make it greater.

*Memoir on the Determination of the Specific Heat of the different Gases.* By MM. F. Delaroche, M.D. and J. E. Berard.*

The subject proposed by the Institute as a prize at the meeting of the 7th January, 1811, namely, the determination of the specific heat of the gases, had previously attracted the attention of different philosophers, some of whom have treated of it in detail; yet so little progress has been made in the investigation, though the question proposed be sufficiently simple, that we are almost as far from being able to answer it with precision, as we were before it became the subject of investigation.

Crawford, as far as we know, is the first person who began the investigation. He published the result of his researches in 1788. At that time a great number of experiments had been made upon the specific heat of bodies in general. MM. Lavoisier and de Laplace had already published the result of their experiments on that subject, and had given a description of their calorimeter of ice; yet Dr. Crawford preferred the method of mixtures of water, or other bodies whose specific heat was considered as known. After many unsuccessful attempts, he thought that he succeeded by the following method. He procured two copper vessels, very thin, and of the same shape, size, and weight. He filled one of them with the gas that he wished to examine, and made a vacuum in the other. He then heated both in boiling water, and plunged both suddenly into cylinders containing a small quantity of cold water, but sufficient to cover them. He subtracted the heat communicated to this water by the exhausted vessel from that communicated by the vessel full of gas, and considered the remainder as the effect produced by the gas, or as its specific heat. He had taken great precautions to ensure the accuracy of his results; but it is quite evident, from the smallness of the change, that no confidence could be placed in them. The rise in the temperature of the water never exceeded 0·4° Fahrenheit. The following is the table of his results:—

| | |
|---|---|
| Specific heat of Water | 1·000 |
| Atmospheric air | 1·790 |
| Oxygen | 4·749 |

* Translated from the Annales de Chimie for January, 1813, vol. lxxxv. p. 72. This memoir gained the prize proposed by the Institute, and deserves particular attention. It overturns the theory of animal heat advanced by Crawford, and Lavoisier's theory of combustion.

Specific

| | | |
|---|---|---|
| Specific heat of | Azote | 0·793 |
| | Carbonic acid | 1·045 |
| | Hydrogen | 21·400 |

Before the work of Crawford appeared, Lavoisier and Laplace had made some experiments, which were not published till long after. They had employed their calorimeter of ice, through which they passed a current of gas contained in a serpentine, which enveloped on all sides the ice of the interior chamber. A thermometer placed at each extremity of the serpentine enabled them to observe the temperature of the gas when it entered and came out of the calorimeter. The gas was heated by passing through a serpentine surrounded with boiling water, before it entered into the calorimeter. These experiments, though susceptible of much greater precision than those of Crawford, were not free from very material imperfections. The method employed by these philosophers to take the temperature of the gases at its entrance into the calorimeter was insufficient, since the gas, in passing through the exterior coating of ice, would lose a portion of the heat which the thermometer had indicated, without contributing by that to melt the ice in the interior chamber. On the other hand, they do not say that they took any precautions to dry the gases upon which they made their experiments. These gases, charged with the humidity which the contact of water in the gazometers would necessarily communicate, no doubt deposited the whole of it when they passed through the calorimeter; but we know that vapor, when it condenses, gives out a great deal of heat. It is proper, however, to remark, that the temperature at which these experiments were made, being probably but little elevated above the freezing point, the quantity of vapor mixed with the gas would not be considerable. Lavoisier and de Laplace only subjected to these experiments oxygen and atmospherical air. They found for the specific heat of the first (that of water being 1·00) 0·65, and for the second 0·33; but Lavoisier acknowledges that the accuracy of these results cannot be entirely depended on.

Since that time, various attempts have been made to appreciate, by indirect means, the specific heat of some gases. Mr. Leslie has employed, in order to compare the specific heat of hydrogen and atmospheric air, a process founded on the following considerations. When a large receiver is partly exhausted of air, if air be allowed to enter into it, the dilated air which it contained will condense, and its temperature will be increased by a constant quantity, whatever gas enter into it; but the entering gas will absorb a part of this excess of heat, and the mixture will have a mean temperature between that of the entering gas and that which the air would have acquired if it had not been obliged to part with any of its heat. Now it is evident that this mean temperature will be so much the lower the greater the specific heat is of the gas which enters. The experiments made by Mr. Leslie have led him to conclude, that equal volumes of hydrogen and atmospherical air have the same specific heat.

The principle upon which this ingenious process is founded is not perfectly just; since it appears from the experiments of Gay-Lussac,* that part of the heat developed in this case comes from the gas that enters into the receiver. It appears, likewise, that some unknown circumstance has misled Mr. Leslie respecting the result of his experiments: for analogous experiments, made with the greatest care, have given different results to Gay-Lussac; and he did not observe that equality of effect which takes place, according to Mr. Leslie, when atmospherical air and hydrogen gas are made to enter into an exhausted receiver. Gay-Lussac, in these experiments, made use of two similar balloons, communicating with each other by a pipe furnished with a stop-cock. He made a vacuum in the one, and filled the other successively with different dried gases. In the centre of each of these balloons was a thermometer of spirit of wine. When the stop-cock was turned, the gas rushed from the full vessel into the empty one. He had taken precautions to render the velocity of the current equal in all cases. The thermometer of the first balloon sunk, and that of the second rose the same number of degrees; but that number varied according to the nature and density of the gases employed. Gay-Lussac, thinking that the specific heat of the gases subjected to these experiments was proportional to the rising and falling of the thermometers, thought himself entitled to conclude that the specific heat of equal volumes of the different gases was inversely as their specific gravity, and of the same gas directly as its density; but he only gave this opinion as a probable conjecture, without affirming any thing respecting its justness. In fact, the phenomena which take place in such cases are very complicated, and it is almost impossible to distinguish what depends upon the different conducting power of the gases from what depends upon their specific heat.

M. Gay-Lussac has discovered this himself, in the new memoir which he has lately published on the subject,* and in which he came to different results. He employed in these last experiments a very simple and ingenious method to de-

* Mem. d'Arcueil, i. 180. † Ann. de Chim. lxxxi. 98.

termine the specific heat of the gases. It consists in passing to the centre of a small reservoir, containing a thermometer, a current of two different gases, the one hot, the other cold. Knowing the temperature of the two gases before their mixture, and that of the mixture, it was easy to infer the ratio between their respective specific heats. This process, besides that it does not give us the ratio between the specific heat of the gases and that of water, is attended with another inconvenience. When only small quantities of gas are employed, a great part of their heat is communicated to the vessels in which the mixture is made, which may lead to erroneous conclusions. Accordingly, as he made his experiments at first on the gases that can be most conveniently procured, such as air, hydrogen, carbonic acid, &c. which, as will be seen afterwards, do not differ much in their capacity for heat, he was induced to believe that the same volumes of all the gases had the same capacity. However, he afterwards published a note,* from which we see that he had brought his process to perfection by operating upon large quantities of gas. By that method he ascertained that hydrogen and carbonic acid have different specific heats, and the numbers which he assigns approach to those which we have ourselves obtained. This induces us to remark to the commissioners, that a first memoir having for its motto *Tectus magis æstuat ignis*, which contains our most important results, was deposited in the hands of the secretary of the Institute on the 3d of February, 1812, more than five months before that note of Gay-Lussac was published in the Annales de Chimie.

Among the attempts made to determine the specific heat of the gases, we ought to reckon the table drawn up by Mr. Dalton from principles purely theoretical, founded on this hypothesis, that the quantities of heat belonging to the ultimate particles of all elastic fluids ought to be the same under the same pressure and at the same temperature. His table is as follows:—

| | | | |
|---|---|---|---|
| Hydrogen gas | 9·382 | Nitrous gas | 0·777 |
| Azotic gas | 1·866 | Oxide of carbon | 0·777 |
| Atmospheric air | 1·759 | Vapor of alcohol | 0·586 |
| Ammonia | 1·555 | Sulphureted hydrogen | 0 583 |
| Olefiant gas | 1·555 | Nitrous oxide gas | 0·549 |
| Oxygen | 1·333 | Vapor of nitric acid | 0·491 |
| Carbureted hydrogen | 1·333 | Carbonic acid | 0·491 |
| Aqueous vapor | 1·[illegible] | Muriatic acid | 0·424 |
| Vapor of ether | 0·848 | | |

* Ann. de Chim. lxxxiii. 106.

Such

Such are the results of the investigation of this subject hitherto made. By comparing them together, it is easy to see how far they differ from one another. Unless we deceive ourselves respecting the justice of those results which we ourselves have obtained, it will be seen, by what follows, how far they all deviate from the truth.

